# Association of clinical consequences of untreated caries in early childhood with oral health-related quality of life

**DOI:** 10.1590/1980-549720260018.supl.1

**Published:** 2026-06-29

**Authors:** Renato Canevari Dutra da Silva, Francine Lorencetti da Silva Campioni, Heloísa Silva Guerra, Fernanda Oliveira Meller, Cleidiane Aparecida de Quadra, Elton Brás Camargo, Antônio Augusto Schäfer

**Affiliations:** I Universidade de Rio Verde, School of Dentistry, Rio Verde Campus - Rio Verde (GO), Brazil.; II Universidade de Rio Verde, School of Medicine, Goiânia Campus - Goiânia, (GO), Brazil.; III Universidade do Extremo Sul Catarinense, Postgraduate Program in Collective Health - Criciúma (SC), Brazil.

**Keywords:** Dental caries, Child, Quality of life, Oral health, Health inequalities

## Abstract

**Objective:**

To evaluate the association between the pulp involvement, ulceration, fistula, abscess (PUFA) index and oral health-related quality of life (OHRQoL) of 5-year-old Brazilian children.

**Methods:**

This was a cross-sectional, population-based study using data from the oral health survey SB Brasil 2023. A total of 7,198 children examined by trained teams were included. The exposure variable was the presence of clinical consequences of untreated caries in primary teeth, assessed by PUFA. OHRQoL was assessed using the SOHO-5 instrument for 5-year-olds. The analysis considered a complex sampling design, using Poisson regression to estimate prevalence ratios (PR) and linear regression.

**Results:**

The prevalence of clinical consequences of untreated caries (PUFA≥1) was 41.1%, with a higher prevalence among Black, Brown, Asian, and Indigenous children compared to White children. Attending school (PR 0.69; 95%CI 0.53-0.90) and a family income above R$ 4,000.00 (PR 0.59; 95%CI 0.42-0.84) showed a protective effect. The North and Central-West regions had higher prevalence rates than the Southeast. and OHRQoL was worse among children of mixed race and indigenous descent compared to those of white skin color. Furthermore, children with dental caries had a 1.44-point higher OHRQoL score (β=1.44; 95%CI 1.01-1.86), which indicates a worse OHRQoL.

**Conclusion:**

The clinical consequences of untreated dental caries have a negative impact on the OHRQoL of Brazilian children, influenced by social and racial determinants. These findings reinforce the need for public policies aimed at reducing regional inequalities and expanding oral health promotion and prevention actions.

## INTRODUCTION

Dental caries remains one of the most prevalent oral diseases in childhood; when left untreated, it can progress to severe clinical conditions such as pulpal involvement, soft tissue ulceration, fistulas, and abscesses, which cause pain, feeding difficulties, and have an impact on the child’s development and quality of life^1^ ^,^ ^2^ ^,^ ^3^. These clinical consequences are not distributed homogeneously throughout the population, being strongly influenced by social, economic, and racial determinants^4^ ^,^ ^5^.

Traditionally, epidemiological surveys regarding oral health have utilized indices such as the DMFT/dmft (decayed, missing, and filled permanent/deciduous teeth), which quantify caries experience but fail to capture its more severe clinical manifestations^6^ ^,^ ^7^ ^,^ ^8^. In this context, the PUFA index (Pulp involvement, Ulceration, Fistula, Abscess) emerges as an essential tool for assessing the clinical consequences of untreated caries in the primary dentition, focusing on lesions that cause pain and infection and, consequently, directly affect the child’s well-being^9^.

By identifying severe lesions, such as pulpal involvement, mucosal ulcerations, fistulas, and abscesses, the PUFA index offers a more detailed overview of the impact of untreated caries^10^. These clinical conditions, associated with acute and chronic pain, difficulty chewing, sleep disturbances, and school absenteeism, impact the child’s general health and behavior^11^. Recent studies have demonstrated that the presence of one or more PUFA lesions is closely linked to pain of dental origin-a symptom that, when present, acts as a direct predictor of poorer oral health-related quality of life (OHRQoL)^12^ ^,^ ^13^ ^,^ ^14^.

OHRQoL assessment in children has been widely conducted using validated instruments such as the scale of oral health outcomes for 5-year-old children (SOHO-5), which measures the impact of oral health on physical, functional, and psychosocial well-being^15^. Research utilizing the SOHO-5 has demonstrated that the accumulation of severe carious lesions, quantified by the PUFA index, is a determinant factor for a negative quality of life score^16^ ^,^ ^17^ ^,^ ^18^. Children with high PUFA scores report more frequently difficulties in eating, speaking, smiling, and engaging socially, as well as a higher incidence of pain and discomfort, thereby corroborating the importance of considering the severe clinical manifestations of dental caries^19^.

The analysis of the clinical consequences of untreated primary tooth caries, via the PUFA index, combined with the assessment of OHRQoL using the SOHO-5, offers a more comprehensive approach to understanding the impact of this disease on children’s lives. The present study aimed to evaluate the association between the clinical consequences of untreated caries (PUFA) and the OHRQoL of 5-year-old Brazilian children.

## METHODS

### Study design and population studied

This is a population-based, cross-sectional study conducted using secondary data from the 2023 National Oral Health Survey (SB Brasil), coordinated by the Ministry of Health. SB Brasil constitutes the most comprehensive epidemiological survey of oral health conducted in the country, featuring national coverage and a multi-stage probabilistic sampling design.

### Population and sampling

The sampling plan for SB Brasil 2023 was designed to ensure national and regional representativeness, covering both state capitals and the interior of Brazilian states. A stratified cluster sample (census tracts) was drawn using a one- or two-stage design; specifically, for the 5-year-old index age group, the sample was obtained in a single stage, meaning that every household within the selected tracts was surveyed to identify potential participants. This study included all participants aged 5 years, totaling 7,198 children.

### Data collection

Data collection was conducted between 2022 and 2023 by field teams comprising dentists and recorders, who had previously undergone a theoretical-practical training and calibration process.

Interviews were conducted with the guardians of 5-year-old children. A structured questionnaire was administered, featuring questions regarding the demographic and socioeconomic profiles of the families and participants, access to and utilization of oral health services, self-reported oral morbidity, self-perception of oral health, and the impact of oral health on daily activities.

The training and calibration process involved lectures, virtual exercises, and in-person activities, including the analysis of photographs and standardized clinical examinations. Inter-examiner agreement relative to a gold standard was assessed using the Kappa coefficient; a minimum value of 0.65 was required for participation in the field.

Clinical examinations were performed in the participants’ homes, utilizing natural light, flat-surface mouth mirrors, and sterilized community periodontal probes, in accordance with World Health Organization (WHO) criteria. Various oral health conditions were assessed, including tooth loss, dental status, dental trauma, and treatment needs, among others.

### Exposure variable

The presence of clinical consequences of untreated caries in primary teeth was assessed using the PUFA index, as employed in the SB Brasil 2023 survey. This index records the clinical consequences of untreated dental caries through four components: pulpal involvement (p), ulceration (u), fistula (f), and abscess (a), as described by Monse et al.^10^ and adopted in the SB Brasil 2020 Technical Project. The assessment was performed individually for each primary tooth examined. In the database, teeth were also assigned the codes “0” (absence of clinical consequences) and “x” (tooth excluded from examination), neither of which is included in the index calculation. For analytical purposes, a dichotomous indicator (PUFA≥1) was constructed: children with at least one tooth classified under any of the four index components (p, u, f, or a) were categorized as presenting clinical consequences of untreated caries (“yes”), while the remainder were categorized as “no”.

### Outcome variable

OHRQoL was assessed using the Brazilian version of the SOHO-5, which has been validated for use in Brazil^20^ and was completed by the children and their parents or guardians. This instrument evaluates the impact of oral health conditions on children’s quality of life, addressing aspects such as physical symptoms, functional limitations, and psychosocial factors. For analytical purposes, the instrument’s total score was calculated by summing the responses to the individual items, with higher scores indicating a greater negative impact of oral health on quality of life. This score was utilized as a continuous variable in the analyses.

### Covariables

The following sociodemographic variables were studied: sex (male, female), skin color (White, Black, Asian, Brown, Indigenous), school attendance (no, yes), maternal schooling (collected in full years and categorized as 0, 1-4, 5-8, 9-11, 12 or more), monthly family income (collected in Reais and categorized as <R$ 1,000; R$ 1,001-R$ 2,000; R$ 3,001-R$ 4,000; >R$ 4,000), macro-region (North, Northeast, Southeast, South, Central-West), municipality (interior, capital), Continuous Cash Benefit (no, yes), receiving Bolsa Família (Family Allowance) (no, yes), benefit from another government program (no, yes), and presence of clinical consequences of untreated caries - PUFA≥1 (no, yes).

### Statistical analysis

Absolute (n) and relative (%) frequencies were presented for all categorical variables studied, as well as their respective 95% confidence intervals (95%CI). The OHRQoL score was presented using the mean and standard deviation, assuming the normality of the data.

Crude and adjusted analyses of the association between the presence of clinical consequences of untreated caries in primary teeth (PUFA≥1) and sociodemographic variables were conducted using Pearson’s χ² test and Poisson regression, respectively; the measure of effect presented was the prevalence ratio (PR) with its respective 95%CI.

Crude and adjusted analyses of the association between the OHRQoL score and sociodemographic variables were presented using means and 95%CIs, and linear regression, respectively; the measure of effect presented was the linear regression coefficient (β) with its respective 95%CI. A significance level of 5% was adopted for all analyses.

The crude and adjusted association between the presence of caries lesions in primary teeth and the OHRQoL score was analyzed using linear regression; the measure of effect presented was the linear regression coefficient (β) and its respective 95%CI. A significance level of 5% was adopted for all analyses.

In the adjusted analyses, three levels of determination were considered:

1. Distal, comprising the variables sex and skin color;
2. Intermediate: including the variables school attendance, maternal education level, monthly family income, and receipt of the Bolsa Família benefit; and
3. Proximal: comprising the variables macro-region and municipality. Variables exhibiting a significance level of 20% (p-value<0.20) remained in the model as potential confounders.

The presence of collinearity between the socioeconomic variables (family income and receiving Bolsa Família benefits) was assessed prior to multivariate analysis using the variance inflation factor (VIF) and the correlation matrix. No values indicative of substantial collinearity were observed, and the variables were retained in the adjusted model. All statistical analyses were performed using Stata version 17.0, incorporating the “svy” prefix to account for the complex sample design.

## Data availability statement

The entire dataset supporting the results of this study is available upon request to the corresponding author. The dataset is not publicly available due to the integration of databases performed by the authors.

## RESULTS

The sociodemographic characteristics of the 5-year-old children are presented in Table 1. The sample consisted of 7,198 children, 51.1% of whom were female, 44.1% self-identified as White, and 44.2% as Brown. The majority attended school (92.5%). The predominant level of maternal schooling was 9 to 11 completed years (44.4%), and family income was concentrated mainly between R$ 1,001 and R$ 2,000 (37.2%). The regional distribution showed a higher concentration in the Northeast (27.8%) and Southeast (38.5%) regions, and the majority resided in capital city areas (78.9%). Regarding socioeconomic status, 42.8% of families received the Bolsa Família benefit, and 16.9% received another government benefit. Finally, the prevalence of clinical consequences of untreated caries (PUFA≥1) was 41.1%.

**Table 1.** Sociodemographic characteristics of the 5-year-old children studied. SB Brasil, 2023 (n=7,198).

Variables	n	% (95%CI)
Sex		
Male	3,677	48.9 (45.9-51.9)
Female	3,521	51.1 (48.1-54.2)
Skin color		
White	2,460	44.1 (39.7-48.5)
Black	739	10.4 (8.2-13.2)
Yellow	77	0.8 (0.5-1.3)
Brown	3,718	44.2 (40.5-48.1)
Indigenous	24	0.4 (0.1-1.6)
School attendance		
No	663	7.5 (5.4-10.3)
Yes	6,457	92.5 (89.7-94.6)
Maternal education (years completed)		
0	554	8.6 (6.6-11.2)
1 to 4	1,189	19.5 (16.6-22.9)
5 to 8	542	8.9 (6.9-11.3)
9 to 11	3,150	44.4 (41.0-47.9)
12 or more	1,115	18.5 (14.4-23.6)
Monthly family income		
<R$ 1,000	812	16.5 (13.3-20.2)
R$ 1,001 to R$ 2,000	1,906	37.2 (32.8-41.8)
R$ 2,001 to R$ 3,000	928	19.0 (16.7-21.4)
R$ 3,001 to R$ 4,000	418	8.9 (7.2-10.9)
>R$ 4,000	741	18.5 (13.8-24.3)
Macro-region		
North	1,771	10.8 (8.9-13.2)
Northeast	2,638	27.8 (23.7-32.2)
Southeast	843	38.5 (32.3-45.1)
South	860	14.3 (11.8-17.1)
Central-West	1,086	8.6 (6.9-10.8)
Municipality		
Interior	1,977	78.9 (75.6-81.8)
Capital	5,221	21.1 (18.2-24.4)
Continuous Cash Benefit		
No	5,953	92.4 (89.8-94.4)
Yes	482	7.6 (5.6-10.2)
Receives Bolsa Família		
No	3,433	57.2 (52.4-61.9)
Yes	3,051	42.8 (38.1-47;6)
Benefit from another government program		
No	5,358	83.1 (80.2-85.7)
Yes	1,083	16.9 (14.3-19.8)
Presence of dental caries in primary teeth		
No	3,979	58.9 (55.3-62.5)
Yes	3,219	41.1 (37.5-44.7)

CI: confidence interval.

After adjusting for potential confounding factors, the presence of clinical consequences of untreated caries was significantly associated with skin color (p<0.001), school attendance (p=0.007), monthly family income (p=0.005), and macro-region (p=0.036). Children self-identifying as Black (PR 1.58; 95%CI 1.23-2.03), Asian (PR 1.96; 95%CI 1.37-2.79), Brown (PR 1.57; 95%CI 1.31-1.87), and Indigenous (PR 2.54; 95%CI 2.06-3.15) presented a higher prevalence of caries compared to children self-reporting as White. School attendance demonstrated a protective effect, with a lower prevalence of clinical consequences among children who attended school (PR 0.69; 95%CI 0.53-0.90). Families with an income exceeding R$ 4,000 showed a 41% lower prevalence of clinical consequences of untreated caries compared to those with an income below R$ 1,000 (PR 0.59; 95%CI 0.42-0.84). Regarding macro-regions, children from the North (PR 1.63; 95%CI 1.29-2.08) and Central-West (PR 1.70; 95%CI 1.33-2.18) regions exhibited a higher prevalence of caries relative to the Southeast region. The other variables studied showed no significant association with the presence of carious lesions in primary teeth (Table 2).

**Table 2.** Crude and adjusted analysis of the association between the presence of clinical consequences of untreated caries (PUFA**≥**1) in primary teeth and the sociodemographic variables studied. SB Brasil, 2023.

Variables	Presence of caries in primary teeth			
	Crude analysis		Adjusted analysis	
	% (95%CI)	p-value*	PR (95%CI)	p-value^†^
Sex				
Male	42.3 (38.6-46.2)	p=0.367	Reference	0.307
Female	39.8 (34.8-45.0)		0.93 (0.81-1.07)	
Skin color				
White	31.0 (26.1-36.4)	p<0.001	Reference	<0.001
Black	49.0 (40.5-57.6)		1.58 (1.23-2.03)	
Yellow	60.7 (40.3-78.0)		1.96 (1.37-2.79)	
Brown	48.6 (44.6-52.8)		1.57 (1.31-1.87)	
Indigenous	78.9 (67.1-87.3)		2.54 (2.06-3.15)	
School attendance				
No	55.4 (44.0-66.2)	p=0.007	Reference	0.007
Yes	39.9 (36.3-43.5)		0.69 (0.53-0.90)	
Maternal education (years completed)				
0	41.3 (32.1-51.1)	p=0.001	Reference	0.311
1 to 4	46.9 (40.6-53.3)		1.28 (0.94-1.74)	
5 to 8	50.5 (37.4-63.6)		1.25 (0.85-1.84)	
9 to 11	40.9 (36.1-45.8)		1.19 (0.89-1.60)	
12 or more	26.7 (21.6-32.6)		0.94 (0.68-1.30)	
Monthly family income				
<R $1,000	52.9 (45.0-60.7)	p<0.001	Reference	0.005
R$ 1,001 to R$ 2,000	43.9 (39.1-48.9)		0.91 (0.74-1.10)	
R$ 2,001 to R$ 3,000	45.2 (38.0-52.6)		1.00 (0.79-1.27)	
R$ 3,001 to R$ 4,000	36.4 (25.8-48.6)		0.86 (0.59-1.25)	
>R$ 4,000	24.6 (17.8-33.0)		0.59 (0.42-0.84)	
Macro-region				
North	57.8 (51.4-63.9)	p<0.001	1.63 (1.29-2.08)	0.036
Northeast	46.9 (42.2-51.7)		1.23 (0.97-1.56)	
Southeast	31.3 (25.1-38.1)		Reference	
South	36.8 (29.9-44.2)		1.29 (0.97-1.71)	
Central-West	52.0 (45.1-58.8)		1.70 (1.33-2.18)	
Municipality				
Interior	41.8 (37.3-46.4)	p=0.165	Reference	0.121
Capital	38.2 (36.0-40.5)		0.91 (0.81-1.03)	
Receives Bolsa Família				
No	33.9 (29.4-38.6)	p<0.001	Reference	0.079
Yes	49.6 (45.3-53.9)		1.16 (0.98-1.36)	

CI: confidence interval; PR: prevalence ratio. *Pearson χ^2^ test. ^†^Poisson regression adjusted for the variables in this table, respecting the hierarchical levels of determination: level 1 (sex, skin color), level 2 (school attendance, maternal education, monthly family income, receives Bolsa Família), and level 3 (macro-region and municipality).

PRs and 95%CIs in bold indicate categories with a statistically significant association, for which the confidence interval does not include the value 1.

The mean OHRQoL score was 0.94 (±1.97), with minimum and maximum values of 0 and 14, respectively (data not presented in a table).

The crude and adjusted associations between OHRQoL and the studied sociodemographic variables are presented in Table 3. It is observed that, after adjusting for potential confounding factors, oral health-related quality of life was associated with skin color (p=0.003) and receipt of the Bolsa Família benefit (<0.001). Children with Brown (β=0.63; 95%CI 0.21-1.05) or Indigenous (β=0.91; 95%CI 0.26-1.57) skin color had higher quality of life scores when compared to those with white skin color. Furthermore, children whose families received Bolsa Família showed higher quality of life scores than those who did not (β=0.41; 95%CI 0.23-0.59). The other variables studied were not associated with OHRQoL.

**Table 3.** Crude and adjusted analysis of the association between oral health-related quality of life (score) and the sociodemographic variables studied. SB Brasil, 2023.

Variables	Oral health-related quality of life		
	Crude analysis	Adjusted analysis	
	Mean (95%CI)	β (95%CI)	p-value*
Sex			
Male	0.94 (0.79-1.09)	Reference	0.536
Female	1.04 (0.68-1;39)	0.09 (-0.20-0.39)	
Skin color			
While	0.69 (0.54-0.84)	Reference	0.003
Black	0.90 (0.64-1.16)	0.21 (-0.06-0.49)	
Yes	1.01 (0.37-1.66)	0.33 (-0.34-0.99)	
Brown	1.32 (0.88-;1.76)	0.63 (0.21-1.05)	
Indigenous	1.60 (0.96-2.24)	0.91 (0.26-1.57)	
School attendance			
No	1.04 (0.69-1.38)	Reference	0.870
Yes	0.99 (0.74-1.24)	0.05 (-0.61-0.72)	
Maternal education (completed years)			
0	0.82 (0.59-1.04)	Reference	0.564
1 to 4	1.29 (0.84-1.74)	0.48 (-0.21-1.18)	
5 to 8	1.80 (1.11-2.50)	0.96 (-0.30-0.44)	
9 to 11	0.88 (0.70-1.06)	0.07 (-0.30-0.44)	
12 or more	0.89 (0.22-1.56)	0.42 (-0.53-1.36)	
Monthly family income (in reals)			
<R$ 1,000	1.39 (1.04-1.75)	Reference	0.331
R$ 1,001 to R$ 2,000	1.15 (0.85-1.46)	-0.07 (-0.56-0.41)	
R$ 2,001 to R$ 3,000	1.36 (0.36-2.36)	0.27 (-0.95-1.48)	
R$ 3,001 to R$ 4,000	0.60 (0.36-0.84)	-0.32 (-0.88-0.24)	
>R$ 4,000	0.61 (0.37-0.85)	-0.28 (-0.98-0.41)	
Receives Bolsa Família			
No	0.78 (0.55-1.01)	Reference	<0.001
Yes	1.30 (0.98-1.62)	0.41 (0.23-0.59)	
Macro-region			
North	0.92 (0.75-1.10)	-0.59 (-1.43-0.24)	0.368
Northeast	0.89 (0.71-1.08)	-0.59 (-1.42-0.24)	
Southeast	1.10 (0.57-1.64)	Referência	
South	0.70 (0.51-0.89)	-0.31 (-0.87-0.25)	
Central-West	1.36 (0.82-1.90)	0.11 (-0.82-1.05)	
Municipality			
Interior	1.01 (0.72-1.29)	Reference	0.596
Capital	0.92 (0.82-1.02)	-0.09 (-0.42-0.24)	

CI: confidence interval; β: linear regression coefficient.

* Linear regression adjusted to the variables in this table, respecting the hierarchical levels of determination: level 1 (sex, skin color), level 2 (school attendance, maternal education, monthly family income, receives Bolsa Família) and level 3 (macro-region and municipality).

p-values in bold indicate global statistical significance of the variable (p<0.05).

β and 95%CI in bold indicate categories com statistically significant association, whose confidence interval does not include the value 0.

**Figure 1.**
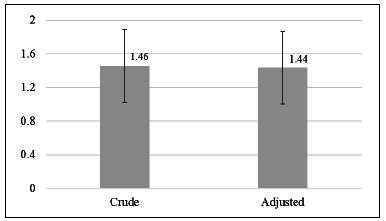
Crude and adjusted analysis of the association between the presence of clinical consequences of untreated caries (PUFA **≥** 1) and oral health-related quality of life (score). SB Brasil, 2023.

Figure 1 displays the crude and adjusted results regarding the association between the presence of carious lesions in primary teeth and oral health-related quality of life. In the crude analysis, children with a PUFA score ≥1 had a quality of life score that was 1.46 points higher than that of children without caries (95%CI 1.02-1.89). After adjustment, the association persisted, albeit with a slight decrease in the effect measure. Children with caries had a quality of life score that was 1.44 points higher when compared to those without caries (95%CI 1.01-1.86).

## DISCUSSION

This study demonstrated that the clinical consequences of early childhood caries, assessed using the PUFA index, are present in more than 40% of 5-year-old Brazilian children and are associated with a negative impact on OHRQoL. This finding reinforces the notion that the experience of caries in the primary dentition transcends the clinical dimension and represents a public health issue characterized by significant social determinants.

Our findings are consistent with data from low- and middle-income countries, where socioeconomic inequalities constitute major barriers to oral health^21^ ^,^ ^22^; conversely, they diverge from the prevalence observed in high-income countries, where the proportion of children with severe carious lesions is less than 15%^23^ ^,^ ^24^. These discrepancies may be attributed to structural differences regarding access to dental services, broader coverage of preventive programs, and well-established public water fluoridation policies in those countries^25^. Thus, the high prevalence of young children presenting with at least one severe clinical consequence of caries represents a concerning statistic and underscores the need to implement policies focused on prevention, surveillance, and access to dental services^26^ ^,^ ^27^.

At the national level, the prevalence observed in this study was high compared to regional investigations. Studies involving Brazilian children reported prevalences of the clinical consequences of dental caries ranging between 12.4 and 65.1% during the preschool years, values ​​lower than those found in the analyzed national sample^28^ ^,^ ^29^ ^,^ ^30^ ^,^ ^31^. In the Northeast region, one study recorded higher rates, indicating regional disparities similar to those observed in this survey^30^. The primary contribution of this work lies in the use of a representative national sample, which allows for the generalization of findings and the comparison of sociodemographic groups and regions, thereby lending greater robustness to the conclusions.

Another relevant finding is that early childhood caries is associated with a poorer quality of life. Children with carious lesions exhibited an OHRQoL score 1.44 points higher than those without the disease, reflecting pain, difficulties with chewing and sleep, as well as psychosocial impact-as evidenced in previous research linking caries to negative outcomes in child development^32^.

The adjusted analysis highlighted social, racial, and territorial inequities, with a higher occurrence of caries in regions characterized by lower levels of collective protection. It is essential to prioritize areas with low water fluoridation and a high disease burden to strengthen school-based initiatives for oral health promotion, to ensure active case-finding and timely care within primary health services, and to monitor for racial equity regarding indicators of access to and receipt of treatment.

Indigenous, Brown (mixed-race), and Black children exhibited a higher prevalence of caries compared to White children. Furthermore, Brown and Indigenous children recorded the poorest OHRQoL scores. These data align with the existing literature regarding structural racism and its impact on health^33^ ^,^ ^34^, suggesting that inequalities in income, educational attainment, access to services, and exposure to risk factors accumulate within racially oppressed groups, ultimately culminating in poorer oral health outcomes. Indeed, inequalities regarding access to restorative treatments have already been documented in the country, with a poorer disease profile and reduced access to restorative care among non-white individuals^35^ ^,^ ^36^. Brazilian studies describe these disparities in both children and adults, emphasizing that part of the variation “by color” actually reflects unequal exposure to collective protective resources and care, rather than biological characteristics^37^ ^,^ ^38^.

Another important finding was that children from higher-income families exhibited a lower prevalence of dental caries. The literature indicates that income influences multiple risk pathways, such as dietary patterns (specifically, a greater availability of ultra-processed foods and sugary beverages in contexts of poverty), the ability to purchase hygiene products, and most importantly timely access to preventive and restorative dental services. Lower income levels and participation in income distribution programs, such as Bolsa Família, were associated with a more negative impact on OHRQoL. Among Brazilian schoolchildren, enrollment in income transfer programs serves to identify vulnerable families; studies have demonstrated a higher prevalence of dental caries and lower utilization of dental services among beneficiaries, even those receiving care through primary health services, thereby underscoring the need for more intensive and proactive strategies for health promotion and care^39^. It is noteworthy that reliance on social benefits, parental employment status, and family income are factors strongly associated with OHRQoL; this association can be attributed to the high cost of dental care, which is less accessible to children from lower socioeconomic background^40^.

The higher prevalence of dental caries observed in the North and Central-West regions, relative to the Southeast, replicates a pattern described in a previous national survey and is linked to regional disparities in the coverage and quality of water fluoridation, as well as in the availability of primary health care services staffed by oral health teams^41^. Furthermore, these disparities reflect a historical pattern of inequality regarding both the provision of health services and the implementation of public policies, such as water fluoridation, manifesting as a higher proportion of water samples falling outside the optimal fluoride range in certain regions, and an observed association between reduced access to fluoridated water and a higher prevalence of untreated dental caries among children^42^ ^,^ ^43^.

In contrast, school attendance demonstrated a significant protective effect, with children attending school exhibiting a lower prevalence of dental caries. This finding suggests that the integration of primary care and schools-through oral health initiatives within the school environment, such as the Saúde na Escola (Health in School) Program, may serve as an effective preventive strategy against dental caries. Technical documents and federal initiatives report the expansion of these activities, including funding for supplies and procedures within public healthcare networks. Although the intensity and quality of these efforts may vary locally, the school’s mediating and protective role regarding hygiene habits, access to preventive care, and timely referral to the healthcare network is noteworthy^44^ ^,^ ^45^.

Certain limitations must be acknowledged, such as the cross-sectional study design, which restricts inferences to associations rather than establishing causal relationships. Furthermore, although the SOHO-5 instrument was administered to both the children and their parents or guardians, it should be noted that, in 5-year-old children, perceptions regarding quality of life may be partially mediated by caregiver reports, potentially introducing perception bias.

A key strength of this study is its focus on a specific age group (5-year-olds), which minimizes variations related to child development. Additionally, the use of a validated instrument to measure OHRQoL and an appropriate methodology for assessing dental caries ensure greater reliability of the results. The integrated analysis of clinical and sociodemographic factors, including participation in income transfer programs, underscores the social relevance of these findings and contributes to a better understanding of the interplay between social inequalities and oral health.

The results of this study demonstrate that the clinical consequences of early childhood caries assessed using the PUFA index negatively impact OHRQoL of 5-year-old Brazilian children. It was observed that these inequalities are complex and multifactorial, involving social, economic, racial, and territorial determinants, with greater vulnerability among Indigenous, multiracial, and Black children, as well as those from low-income families or residing in regions with lower coverage of dental services. Conversely, school attendance demonstrated a protective effect, indicating that integrating primary care with oral health initiatives within the school environment can reduce the burden of disease.

In this context, two structural strategies within the Unified Health System (SUS) merit particular attention in addressing the oral health inequities observed in this study. The fluoridation of public water supplies, recognized as one of the most cost-effective measures for preventing dental caries, has a significant impact on reducing the disease, particularly among children from more socioeconomically vulnerable groups^45^ ^,^ ^46^. National evidence indicates that discontinuities in fluoridation and failures in monitoring fluoride levels contribute to the persistence of regional inequalities and to higher levels of untreated caries during childhood^47^.

Furthermore, the proper organization of primary health care, specifically through the effective integration of Oral Health Teams (eSB) into the Family Health Strategy, plays a central role in promoting oral health equity^48^. However, in many Brazilian municipalities, an inadequate ratio between family health teams and eSBs is observed; this overburdens the work of the eSBs and limits the reach of preventive and educational initiatives, particularly among preschool-aged children. These organizational weaknesses compromise the capacity of primary care to address, in a timely manner, the social determinants of dental caries and its more severe clinical consequences.

Thus, strengthening water fluoridation through adequate monitoring, combined with expanding and enhancing the work of eSBs within the Family Health Strategy, proves essential for reducing the clinical consequences of caries in early childhood and its impact on OHRQoL^47^ ^,^ ^48^. These findings underscore that reducing oral health inequalities depends on consolidating structural public policies capable of addressing territorial and social vulnerabilities from the very first years of life.
