# Supplementary material for: Association of clinical consequences of untreated caries in early childhood with oral health-related quality of life
Source: Rev Bras Epidemiol. 2026 Jun 29;29(Suppl 1):e260018supl1. doi: 10.1590/1980-549720260018.supl.1 (PMC13325561; doi:10.1590/1980-549720260018.supl.1)
Supplement: Translation

# Translation: Associação das consequências clínicas da cárie na primeira infância e qualidade de vida relacionada à saúde bucal

**DOI:** 10.1590/1980-549720260018.supl.1.2

**Authors:** Renato Canevari Dutra da Silva, Francine Lorencetti da Silva Campioni, Heloísa Silva Guerra, Fernanda Oliveira Meller, Cleidiane Aparecida de Quadra, Elton Brás Camargo, Antônio Augusto Schäfer

**Affiliations:** I Universidade de Rio Verde, Faculdade de Odontologia do Campus Rio Verde - Rio Verde (GO), Brasil.; II Universidade de Rio Verde, Faculdade de Medicina do Campus Goiânia - Goiânia, (GO), Brasil.; III Universidade do Extremo Sul Catarinense, Programa de Pós-Graduação em Saúde Coletiva - Criciúma (SC), Brasil.

**Keywords:** Cárie dentária, Criança, Qualidade de vida, Saúde bucal, Desigualdades em saúde

## Abstract

**Objetivo:**

Avaliar a associação entre Envolvimento Pulpar, Ulceração, Fístula e Abscesso (PUFA) e Qualidade de Vida Relacionada à Saúde Bucal (QVRSB) de crianças brasileiras de 5 anos de idade.

**Métodos:**

Estudo transversal, de base populacional, utilizando dados da Pesquisa Nacional de Saúde Bucal (SB Brasil) 2023. Foram incluídas 7.198 crianças examinadas por equipes treinadas. A variável de exposição foi a presença de consequências clínicas da cárie não tratada em dentes decíduos, avaliada pelo índice PUFA. A QVRSB foi avaliada pelo instrumento *Scale of Oral Health Outcomes for 5-year-old children* (SOHO-5). As análises consideraram o delineamento amostral complexo, utilizando regressão de Poisson para estimar Razões de Prevalência (RP) e regressão linear.

**Resultados:**

A prevalência de consequências clínicas da cárie não tratada (PUFA≥1) foi de 41,1%, sendo maior entre crianças negras, pardas, amarelas e indígenas, quando comparadas às brancas. Frequentar a escola (RP 0,69; IC95% 0,53-0,90) e renda familiar acima de R$ 4.000 (RP 0,59; IC95% 0,42-0,84) mostraram efeito protetor. As regiões Norte e Centro-Oeste apresentaram maiores prevalências em relação à Sudeste. A qualidade de vida relacionada à saúde bucal foi pior entre crianças pardas e indígenas comparadas às de cor de pele branca. Além disso, crianças com cárie tiveram 1,44 ponto a mais no escore de QVRSB (β=1,44; IC95% 1,01-1,86), o que significa pior QVRSB.

**Conclusão:**

As consequências clínicas da cárie dentária não tratada trazem impacto negativo na QVRSB de crianças brasileiras, sendo influenciada por determinantes sociais e raciais. Os achados reforçam a necessidade de políticas públicas voltadas à redução das desigualdades regionais e ampliação das ações de promoção e prevenção em saúde bucal.

## INTRODUÇÃO

A cárie dentária ainda é uma das doenças bucais mais prevalentes na infância e, quando não tratada, pode evoluir para condições clínicas graves como envolvimento pulpar, ulceração de tecidos moles, fístulas e abscessos, que provocam dor, dificuldades alimentares e impacto no desenvolvimento e na qualidade de vida da criança^1^ ^,^ ^2^ ^,^ ^3^. Essas consequências clínicas não se distribuem de forma homogênea na população, sendo fortemente influenciadas por determinantes sociais, econômicos e raciais^4^ ^,^ ^5^.

Tradicionalmente, levantamentos epidemiológicos relacionados à saúde bucal utilizam índices como o CPO-D/ceo-d (dentes permanentes/decíduos cariados, perdidos e obturados), que quantificam a experiência de cárie, mas não capturam suas manifestações clínicas mais graves^6^ ^,^ ^7^ ^,^ ^8^. Nesse contexto, o índice PUFA (*Pulp involvement, Ulceration, Fistula, Abscess*) surge como uma ferramenta essencial para avaliar as consequências clínicas da cárie não tratada na dentição decídua, focando nas lesões que causam dor e infecção e, consequentemente, afetam diretamente o bem-estar da criança^9^.

O índice PUFA, ao identificar lesões graves como envolvimento pulpar, ulcerações da mucosa, fístulas e abscessos, oferece um panorama mais detalhado do impacto da cárie não tratada^10^. Essas condições clínicas, associadas a dor aguda e crônica, dificuldade para mastigar, distúrbios do sono e absenteísmo escolar, impactam a saúde geral e o comportamento da criança^11^. Estudos recentes têm demonstrado que a presença de uma ou mais lesões PUFA está intimamente ligada à dor de origem dentária, um sintoma que, quando presente, atua como um preditor direto de uma pior Qualidade de Vida Relacionada à Saúde Bucal (QVRSB)^12^ ^,^ ^13^ ^,^ ^14^.

A avaliação da QVRSB em crianças tem sido amplamente realizada por meio de instrumentos validados como o *Scale of Oral Health Outcomes for 5-year-old children* (SOHO-5), que mensura o impacto da saúde bucal no bem-estar físico, funcional e psicossocial^15^. Pesquisas utilizando o SOHO-5 têm evidenciado que o acúmulo de lesões graves de cárie, quantificadas pelo índice PUFA, é um fator determinante para um escore negativo na qualidade de vida^16^ ^,^ ^17^ ^,^ ^18^. Crianças com altos valores de PUFA relatam mais frequentemente dificuldades para comer, falar, sorrir e se relacionar socialmente, além de maior incidência de dor e desconforto, corroborando a importância de se considerar as manifestações clínicas graves da cárie^19^.

A análise das consequências clínicas da cárie dentária decídua não tratada, por meio do índice PUFA, em conjunto com a avaliação da QVRSB pelo SOHO-5, oferece uma abordagem mais completa para compreender o impacto da doença na vida das crianças. O presente estudo teve como objetivo avaliar a associação entre as consequências clínicas da cárie não tratada (PUFA) e a QVRSB de crianças brasileiras de 5 anos de idade.

## MÉTODOS

### Desenho do estudo e população estudada

Trata-se de um estudo transversal, de base populacional, realizado com dados secundários da Pesquisa Nacional de Saúde Bucal (SB Brasil) 2023, coordenada pelo Ministério da Saúde. O SB Brasil constitui o mais abrangente inquérito epidemiológico de saúde bucal realizado no país, com abrangência nacional e desenho amostral probabilístico em múltiplos estágios.

### População e amostragem

O plano amostral do SB Brasil 2023 foi delineado para garantir representatividade nacional, regional, por capitais e interior dos estados brasileiros. Foi sorteada amostra estratificada por conglomerados (setores censitários) em um ou dois estágios, sendo que para a idade-índice 5 anos a amostra foi obtida em estágio único, ou seja, todas as residências dos setores sorteados foram pesquisadas para busca de potenciais participantes. Neste estudo, foram incluídos todos os participantes com 5 anos de idade, totalizando 7.198 crianças.

### Coleta de dados

A coleta foi realizada entre 2022 e 2023 por equipes de campo formadas por cirurgiões-dentistas e anotadores, previamente submetidos a processo de treinamento teórico-prático e calibração.

As entrevistas foram conduzidas com os responsáveis das crianças de 5 anos de idade. Um questionário estruturado foi aplicado com questões sobre o perfil demográfico e socioeconômico das famílias e dos participantes, acesso e utilização de serviços de saúde bucal, morbidade bucal referida, autopercepção de saúde bucal e impacto da saúde bucal nas atividades diárias.

O treinamento e a calibração envolveram aulas, exercícios em ambiente virtual e atividades presenciais, com análise de fotografias e exames clínicos padronizados. A concordância entre examinadores e padrão-ouro foi avaliada por meio do coeficiente Kappa, exigindo-se valores mínimos de 0,65 para participação no campo.

Os exames clínicos foram realizados nos domicílios, com iluminação natural, utilizando espelhos bucais planos e sondas comunitárias esterilizadas, seguindo os critérios da Organização Mundial da Saúde (OMS). Foram avaliados diferentes agravos bucais, incluindo perda dentária, condição dentária (CPO-D/ceo-d), traumatismos, necessidade de tratamento, entre outros.

### Variável de exposição

A presença de consequências clínicas da cárie não tratada em dentes decíduos foi avaliada mediante a aplicação do índice PUFA e utilizado no SB Brasil 2023. Esse índice registra as consequências clínicas da cárie dentária não tratada por meio de quatro componentes: envolvimento pulpar (p), ulceração (u), fístula (f) e abscesso (a), conforme descrito por Monse et al.^10^ e adotado no Projeto Técnico do SB Brasil 2020. A avaliação foi realizada individualmente em cada dente decíduo examinado. No banco de dados, os dentes também receberam os códigos “0” (ausência de consequências clínicas) e “x” (dente excluído do exame), que não integram o cálculo do índice. Para fins de análise, foi construído um indicador dicotômico (PUFA≥1): crianças com pelo menos um dente classificado em qualquer um dos quatro componentes do índice (p, u, f ou a) foram categorizadas como tendo presença de consequências clínicas da cárie não tratada (“sim”), enquanto as demais foram categorizadas como “não”.

### Variável de desfecho

A qualidade de vida relacionada à saúde bucal foi avaliada por meio da versão brasileira da SOHO-5, validada para uso no Brasil^20^ e que foi respondida pelas crianças e por seus pais ou responsáveis. O instrumento avalia o impacto das condições de saúde bucal na qualidade de vida de crianças, abordando aspectos como sintomas físicos, limitações funcionais e aspectos psicossociais. Para fins analíticos, foi calculado o escore total do instrumento por meio da soma das respostas dos itens, sendo que escores mais elevados indicam maior impacto negativo da saúde bucal na qualidade de vida. O escore foi utilizado como variável contínua nas análises.

### Covariáveis

Foram estudadas as variáveis sociodemográficas: sexo (masculino, feminino), cor da pele (branca, preta, amarela, parda, indígena), frequenta a escola (não, sim), escolaridade materna (coletada em anos completos e categorizada em 0, 1-4, 5-8, 9-11, 12 ou mais), renda familiar mensal (coletada em reais e categorizada em <R$1.000; R$1.001-R$2.000; R$3.001-R$4.000; >R$4.000), macrorregião (Norte, Nordeste, Sudeste, Sul, Centro-Oeste), município (interior, capital), benefício assistencial de prestação continuada (não, sim), recebimento de Bolsa Família (não, sim), benefício de outro programa do governo (não, sim), presença de consequências clínicas da cárie não tratada - PUFA≥1 (não, sim).

### Análise estatística

Foram apresentadas as frequências absolutas (n) e relativas (%) de todas as variáveis categóricas estudadas bem como seus respectivos Intervalos de Confiança de 95% (IC95%). O escore da QVRSB foi apresentado por meio de média e desvio-padrão considerando a normalidade dos dados.

A análise bruta e ajustada da associação entre presença de consequências clínicas da cárie não tratada em dentes decíduos (PUFA≥1) e as variáveis sociodemográficas foi conduzida, respectivamente, por meio do teste χ^2^ de Pearson e da regressão de Poisson, apresentando como medida de efeito a Razão de Prevalência (RP) com seu respectivo IC95%.

Análise bruta e ajustada da associação entre escore de QVRSB e as variáveis sociodemográficas foram apresentadas, respectivamente, por médias, IC95% e regressão linear, apresentando como medida de efeito o coeficiente de regressão linear (β) com seu respectivo IC95%. Para todas as análises foi considerado nível de significância de 5%.

A associação bruta e ajustada entre a presença de lesões de cárie em dentes decíduos e o escore de QVRSB foi analisada por meio da regressão linear, apresentando como medida de efeito o coeficiente de regressão linear (β) e seu respectivo IC 95%. Para todas as análises foi considerado nível de significância de 5%.

Nas análises ajustadas, consideraram-se três níveis de determinação, sendo eles:

1. Distal, contendo as variáveis sexo e cor da pele;
2. Intermediário, incluindo as variáveis frequenta a escola, escolaridade materna, renda familiar mensal e recebimento de Bolsa Família; e
3. Proximal, com as variáveis macrorregião e município. Permaneceram no modelo como possíveis fatores de confusão as variáveis que apresentaram nível de significância de 20% (valor p<0,20).

A presença de colinearidade entre as variáveis socioeconômicas (renda familiar e recebimento do Bolsa Família) foi avaliada previamente à análise multivariada por meio do Fator de Inflação da Variância (VIF) e da matriz de correlação. Não foram observados valores indicativos de colinearidade relevante, e as variáveis foram mantidas no modelo ajustado. Para todas as análises estatísticas utilizou-se o programa *Stata* versão 17.0, considerando o prefixo “svy” devido à complexidade amostral.

## Declaração de disponibilidade de dados

Todo o conjunto de dados que dá suporte aos resultados deste estudo está disponível mediante solicitação ao autor correspondente. O conjunto de dados não está publicamente disponível devido à integração de bancos de dados realizados pelos autores.

## RESULTADOS

As características sociodemográficas das crianças de 5 anos estão apresentadas na Tabela 1. A amostra foi composta por 7.198 crianças, sendo 51,1% do sexo feminino, 44,1% de cor de pele branca e 44,2%, parda. A maioria frequentava a escola (92,5%). A escolaridade materna predominante foi de 9 a 11 anos completos (44,4%), e a renda familiar concentrou-se principalmente entre R$1.001 e R$2.000 (37,2%). A distribuição regional mostrou maior concentração no Nordeste (27,8%) e Sudeste (38,5%), e a maioria residia em áreas de capital (78,9%). Quanto à situação socioeconômica, 42,8% das famílias recebiam Bolsa Família e 16,9%, outro benefício governamental. Por fim, a prevalência de consequências clínicas da cárie não tratada (PUFA≥1) foi de 41,1%.

**Tabela 1.** Características sociodemográficas das crianças de 5 anos de idade estudadas. SB Brasil, 2023 (n=7.198).

Variáveis	n	% (IC95%)
Sexo		
Masculino	3.677	48,9 (45,9-51,9)
Feminino	3.521	51,1 (48,1-54,2)
Cor da pele		
Branca	2.460	44,1 (39,7-48,5)
Preta	739	10,4 (8,2-13,2)
Amarela	77	0,8 (0,5-1,3)
Parda	3.718	44,2 (40,5-48,1)
Indígena	24	0,4 (0,1-1,6)
Frequenta a escola		
Não	663	7,5 (5,4-10,3)
Sim	6.457	92,5 (89,7-94,6)
Escolaridade materna (anos completos)		
0	554	8,6 (6,6-11,2)
1 a 4	1.189	19,5 (16,6-22,9)
5 a 8	542	8,9 (6,9-11,3)
9 a 11	3.150	44,4 (41,0-47,9)
12 ou mais	1.115	18,5 (14,4-23,6)
Renda familiar mensal		
<R$1.000	812	16,5 (13,3-20,2)
R$1.001 a R$2.000	1.906	37,2 (32,8-41,8)
R$2.001 a R$3.000	928	19,0 (16,7-21,4)
R$3.001 a R$4.000	418	8,9 (7,2-10,9)
>R$ 4.000	741	18,5 (13,8-24,3)
Macrorregião		
Norte	1.771	10,8 (8,9-13,2)
Nordeste	2.638	27,8 (23,7-32,2)
Sudeste	843	38,5 (32,3-45,1)
Sul	860	14,3 (11,8-17,1)
Centro-Oeste	1.086	8,6 (6,9-10,8)
Município		
Interior	1.977	78,9 (75,6-81,8)
Capital	5.221	21,1 (18,2-24,4)
Benefício assistencial de prestação continuada		
Não	5.953	92,4 (89,8-94,4)
Sim	482	7,6 (5,6-10,2)
Recebimento de Bolsa Família		
Não	3.433	57,2 (52,4-61,9)
Sim	3.051	42,8 (38,1-47;6)
Benefício de outro programa do governo		
Não	5.358	83,1 (80,2-85,7)
Sim	1.083	16,9 (14,3-19,8)
Presença de lesões de cárie em dentes decíduos		
Não	3.979	58,9 (55,3-62,5)
Sim	3.219	41,1 (37,5-44,7)

IC: intervalo de confiança.

Após ajuste para possíveis fatores de confusão, a presença de consequências clínicas da cárie não tratada esteve significativamente associada à cor da pele (p<0,001), frequência escolar (p=0,007), renda familiar mensal (p=0,005) e macrorregião (p=0,036). Crianças de cor de pele preta (RP 1,58; IC95% 1,23-2,03), amarela (RP 1,96; IC95% 1,37-2,79), parda (RP 1,57; IC95% 1,31-1,87) e indígena (RP 2,54; IC95% 2,06-3,15) apresentaram maior prevalência de lesões de cárie em relação às crianças de cor de pele branca. A frequência escolar apresentou efeito protetor, com menor prevalência de consequências clínicas entre crianças que frequentavam a escola (RP 0,69; IC95% 0,53-0,90). Famílias com renda superior a R$4.000 apresentaram prevalência 41% menor de consequências clínicas da cárie não tratada em comparação às com renda inferior a R$1.000 (RP 0,59; IC95% 0,42-0,84). Quanto à macrorregião, as crianças do Norte (RP 1,63; IC95% 1,29-2,08) e Centro-Oeste (RP 1,70; IC95% 1,33-2,18) apresentaram maior prevalência de cárie em relação à região Sudeste. As demais variáveis estudadas não apresentaram associação significativa com a presença de lesões de cárie em dentes decíduos (Tabela 2).

**Tabela 2.** Análise bruta e ajustada da associação entre presença de consequências clínicas da cárie não tratada (PUFA**≥**1) em dentes decíduos e as variáveis sociodemográficas estudadas. SB Brasil, 2023.

Variáveis	Presença de lesões de cárie em dentes decíduos			
	Análise bruta		Análise ajustada	
	% (IC95%)	Valor de p*	RP (IC95%)	Valor de p^†^
Sexo				
Masculino	42,3 (38,6-46,2)	p=0,367	Referência	0,307
Feminino	39,8 (34,8-45,0)		0,93 (0,81-1,07)	
Cor da pele				
Branca	31,0 (26,1-36,4)	p<0,001	Referência	<0,001
Preta	49,0 (40,5-57,6)		1,58 (1,23-2,03)	
Amarela	60,7 (40,3-78,0)		1,96 (1,37-2,79)	
Parda	48,6 (44,6-52,8)		1,57 (1,31-1,87)	
Indígena	78,9 (67,1-87,3)		2,54 (2,06-3,15)	
Frequenta a escola				
Não	55,4 (44,0-66,2)	p=0,007	Referência	0,007
Sim	39,9 (36,3-43,5)		0,69 (0,53-0,90)	
Escolaridade materna (anos completos)				
0	41,3 (32,1-51,1)	p=0,001	Referência	0,311
1 a 4	46,9 (40,6-53,3)		1,28 (0,94-1,74)	
5 a 8	50,5 (37,4-63,6)		1,25 (0,85-1,84)	
9 a 11	40,9 (36,1-45,8)		1,19 (0,89-1,60)	
12 ou mais	26,7 (21,6-32,6)		0,94 (0,68-1,30)	
Renda familiar mensal				
<R$1.000	52,9 (45,0-60,7)	p<0,001	Referência	0,005
R$1.001 a R$2.000	43,9 (39,1-48,9)		0,91 (0,74-1,10)	
R$2.001 a R$3.000	45,2 (38,0-52,6)		1,00 (0,79-1,27)	
R$3.001 a R$4.000	36,4 (25,8-48,6)		0,86 (0,59-1,25)	
>R$4.000	24,6 (17,8-33,0)		0,59 (0,42-0,84)	
Macrorregião				
Norte	57,8 (51,4-63,9)	p<0,001	1,63 (1,29-2,08)	0,036
Nordeste	46,9 (42,2-51,7)		1,23 (0,97-1,56)	
Sudeste	31,3 (25,1-38,1)		Referência	
Sul	36,8 (29,9-44,2)		1,29 (0,97-1,71)	
Centro-Oeste	52,0 (45,1-58,8)		1,70 (1,33-2,18)	
Município				
Interior	41,8 (37,3-46,4)	p=0,165	Referência	0,121
Capital	38,2 (36,0-40,5)		0,91 (0,81-1,03)	
Recebimento de Bolsa Família				
Não	33,9 (29,4-38,6)	p<0,001	Referência	0,079
Sim	49,6 (45,3-53,9)		1,16 (0,98-1,36)	

IC: intervalo de confiança; RP: razão de prevalência.

*Teste χ^2^ de Pearson. ^†^Regressão de Poisson ajustada para as variáveis dessa tabela respeitando os níveis hierárquicos de determinação: nível 1 (sexo, cor da pele), nível 2 (frequenta a escola, escolaridade materna, renda familiar mensal, recebimento de Bolsa Família) e nível 3 (macrorregião e município).

Valores de p em negrito indicam significância estatística global da variável (p<0,05).

RP e IC 95% em negrito indicam categorias com associação estatisticamente significativa, cujo intervalo de confiança não inclui o valor 1.

A média do escore de QVRSB foi de 0,94 (±1,97), com valores mínimo e máximo de 0 a 14, respectivamente (dados não apresentados em tabela).

A associação bruta e ajustada entre a QVRSB e as variáveis sociodemográficas estudadas é apresentada na Tabela 3. Observa-se que, após ajuste para possíveis fatores de confusão, a qualidade de vida relacionada à saúde bucal esteve relacionada com cor da pele (p=0,003) e recebimento de Bolsa Família (<0,001). Crianças de cor de pele parda (β:=0,63; IC95% 0,21-1,05) ou indígenas (β=0,91; IC95% 0,26-1,57) tiveram maiores escores de qualidade de vida quando comparadas às de cor de pele branca. Além disso, aquelas crianças cujas famílias recebiam Bolsa Família apresentaram maior escore de qualidade de vida do que as que não recebiam (β=0,41; IC95% 0,23-0,59). As demais variáveis estudadas não estiveram associadas com a QVRSB.

**Tabela 3.** Análise bruta e ajustada da associação entre qualidade de vida relacionada à saúde bucal (escore) e as variáveis sociodemográficas estudadas. SB Brasil, 2023.

Variáveis	Qualidade de vida relacionada à saúde bucal		
	Análise bruta	Análise ajustada	
	Média (IC95%)	β (IC95%)	Valor de p*
Sexo			
Masculino	0,94 (0,79-1,09)	Referência	0,536
Feminino	1,04 (0,68-1;39)	0,09 (-0,20-0,39)	
Cor da pele			
Branca	0,69 (0,54-0,84)	Referência	0,003
Preta	0,90 (0,64-1,16)	0,21 (-0,06-0,49)	
Amarela	1,01 (0,37-1,66)	0,33 (-0,34-0,99)	
Parda	1,32 (0,88-;1,76)	0,63 (0,21-1,05)	
Indígena	1,60 (0,96-2,24)	0,91 (0,26-1,57)	
Frequenta a escola			
Não	1,04 (0,69-1,38)	Referência	0,870
Sim	0,99 (0,74-1,24)	0,05 (-0,61-0,72)	
Escolaridade materna (anos completos)			
0	0,82 (0,59-1,04)	Referência	0,564
1 a 4	1,29 (0,84-1,74)	0,48 (-0,21-1,18)	
5 a 8	1,80 (1,11-2,50)	0,96 (-0,30-0,44)	
9 a 11	0,88 (0,70-1,06)	0,07 (-0,30-0,44)	
12 ou mais	0,89 (0,22-1,56)	0,42 (-0,53-1,36)	
Renda familiar mensal (em reais)			
<R$1.000	1,39 (1,04-1,75)	Referência	0,331
R$1.001 a R$2.000	1,15 (0,85-1,46)	-0,07 (-0,56-0,41)	
R$2.001 a R$3.000	1,36 (0,36-2,36)	0,27 (-0,95-1,48)	
R$3.001 a R$4.000	0,60 (0,36-0,84)	-0,32 (-0,88-0,24)	
>R$4.000	0,61 (0,37-0,85)	-0,28 (-0,98-0,41)	
Recebimento de Bolsa Família			
Não	0,78 (0,55-1,01)	Referência	<0,001
Sim	1,30 (0,98-1,62)	0,41 (0,23-0,59)	
Macrorregião			
Norte	0,92 (0,75-1,10)	-0,59 (-1,43-0,24)	0,368
Nordeste	0,89 (0,71-1,08)	-0,59 (-1,42-0,24)	
Sudeste	1,10 (0,57-1,64)	Referência	
Sul	0,70 (0,51-0,89)	-0,31 (-0,87-0,25)	
Centro-Oeste	1,36 (0,82-1,90)	0,11 (-0,82-1,05)	
Município			
Interior	1,01 (0,72-1,29)	Referência	0,596
Capital	0,92 (0,82-1,02)	-0,09 (-0,42-0,24)	

IC: intervalo de confiança; β: coeficiente de regressão linear.

*Regressão linear ajustada para as variáveis dessa tabela respeitando os níveis hierárquicos de determinação: nível 1 (sexo, cor da pele), nível 2 (frequenta a escola, escolaridade materna, renda familiar mensal, recebimento de Bolsa Família) e nível 3 (macrorregião e município).

Valores de p em negrito indicam significância estatística global da variável (p<0,05).

β e IC 95% em negrito indicam categorias com associação estatisticamente significativa, cujo intervalo de confiança não inclui o valor 0.

A Figura 1 mostra os resultados brutos e ajustados da associação entre presença de lesões de cárie em dentes decíduos e qualidade de vida relacionada à saúde bucal. Na análise bruta, crianças com PUFA≥1 tiveram 1,46 ponto a mais no escore de qualidade de vida do que aquelas sem cárie (IC95% 1,02-1,89). Após ajuste, a associação permaneceu, com uma discreta diminuição na medida de efeito. Aquelas crianças com cárie tiveram 1,44 ponto a mais no escore de qualidade de vida quando comparadas às que não apresentavam cárie (IC95% 1,01-1,86).

**Figura 1.** Análise bruta e ajustada da associação entre presença de consequências clínicas da cárie não tratada (PUFA**≥**1) e qualidade de vida relacionada à saúde bucal (escore). SB Brasil, 2023.

## DISCUSSÃO

Este estudo evidenciou que as consequências clínicas da cárie dentária na primeira infância, avaliadas pelo índice PUFA, estão presentes em mais de 40% das crianças brasileiras de 5 anos e se associam a um impacto negativo na QVRSB. Tal achado reforça que a experiência de cárie na dentição decídua transcende a dimensão clínica e representa um problema de saúde pública com determinantes sociais marcantes.

Nossos achados são consistentes com dados de países de baixa e média renda, onde as desigualdades socioeconômicas são barreiras importantes para a saúde bucal^21^ ^,^ ^22^ e divergem da prevalência observada em países de alta renda, onde a proporção de crianças com lesões de cárie severa é inferior a 15%^23^ ^,^ ^24^. Essas discrepâncias podem estar relacionadas a diferenças estruturais no acesso aos serviços odontológicos, maior cobertura de programas preventivos e políticas públicas consolidadas de fluoretação da água nesses países^25^. Assim, a alta prevalência de crianças na primeira infância com pelo menos uma consequência clínica severa de cárie revela um dado preocupante e reforça a necessidade de implementação de políticas de prevenção, vigilância e acesso a serviços odontológicos^26^ ^,^ ^27^.

Em nível nacional, a prevalência observada neste estudo foi elevada em comparação a investigações regionais. Estudos com crianças brasileiras relataram prevalências de consequências clínicas da cárie entre 12,4 e 65,1% na idade pré-escolar, valores inferiores aos encontrados na amostra nacional analisada^28^ ^,^ ^29^ ^,^ ^30^ ^,^ ^31^. No Nordeste, um estudo registrou taxas mais altas, indicando disparidades regionais semelhantes às observadas neste levantamento^30^. A principal contribuição deste trabalho é o uso de uma amostra nacional representativa, que permite generalizar os achados e comparar grupos sociodemográficos e regiões, conferindo maior robustez às conclusões.

Outro achado relevante é que a cárie na primeira infância está associada a pior qualidade de vida. Crianças com lesões de cárie apresentaram escore de QVRSB 1,44 ponto maior do que aquelas sem a doença, refletindo dor, dificuldades de mastigação e sono, além de impacto psicossocial, conforme evidenciado em pesquisas anteriores que relacionam a cárie a desfechos negativos no desenvolvimento infantil^32^.

A análise ajustada evidenciou iniquidades sociais, raciais e territoriais, com maior ocorrência de cárie em regiões com menor proteção coletiva. É essencial priorizar áreas com baixa fluoretação e alta carga de doença, fortalecer ações escolares de promoção da saúde bucal, garantir busca ativa e cuidado oportuno na atenção primária e monitorar a equidade racial nos indicadores de acesso e tratamento.

Crianças indígenas, pardas e pretas apresentaram maior prevalência de cárie em comparação às brancas. Adicionalmente, crianças pardas e indígenas tiveram os piores escores de QVRSB. Esses dados convergem com a literatura no que concerne ao racismo estrutural e seus impactos na saúde^33^ ^,^ ^34^, o que pode sugerir que as desigualdades em renda, escolaridade, acesso a serviços e exposição a fatores de risco se acumulam em grupos racialmente oprimidos e culminam em piores desfechos relacionados à saúde bucal. De fato, desigualdades quanto ao acesso a tratamentos restauradores já foram documentadas no país, com pior perfil de doença e menor acesso a tratamento restaurador entre pessoas não brancas^35^ ^,^ ^36^. Estudos brasileiros descrevem esses diferenciais tanto em crianças quanto em adultos e reforçam que parte da variação “por cor”, na verdade, reflete exposição desigual a bens de proteção coletiva e cuidados, não características biológicas^37^ ^,^ ^38^.

Outro ponto importante foi que crianças de famílias com maior renda apresentaram menor prevalência de cárie. A literatura mostra que a renda influencia múltiplos caminhos de risco, como o padrão alimentar (maior oferta de ultraprocessados e bebidas açucaradas em contextos de pobreza), possibilidade de adquirir insumos de higiene e, principalmente, acesso a serviços odontológicos preventivos e restauradores no tempo oportuno. Renda mais baixa e participação em programas de distribuição de renda, como o Bolsa Família, indicaram impacto mais negativo na QVRSB. Entre escolares brasileiros, a vinculação a programas de transferência de renda permite identificar famílias vulneráveis, demonstrando maior experiência de cárie e menor uso de serviços entre beneficiários, mesmo acompanhados pela atenção primária, o que reforça a necessidade de estratégias mais intensivas e proativas de promoção e cuidado^39^. Destaca-se que a dependência de benefícios, o emprego paterno e a renda familiar são fatores fortemente associados à QVRSB, o que pode ser explicado pelos altos custos do cuidado odontológico, menos acessível a crianças em menor posição socioeconômica^40^.

A maior prevalência de cárie nas regiões Norte e Centro-Oeste, em relação ao Sudeste, reproduz um padrão descrito em inquérito nacional anterior e guarda relação com diferenças na cobertura e qualidade da fluoretação das águas e na oferta de atenção primária com equipe de saúde bucal^41^. Essas disparidades refletem, ainda, um padrão histórico de desigualdades na oferta de serviços de saúde e na implementação de políticas públicas, como a fluoretação das águas de abastecimento, com maior proporção de amostras fora da faixa ideal em determinadas regiões; e associação entre menor acesso à água fluoretada e maior cárie não tratada em crianças^42^ ^,^ ^43^.

Em contraponto, a frequência escolar demonstrou efeito protetor significativo, com crianças que frequentam a escola apresentando menor prevalência de cárie. Esse achado sugere que a articulação entre atenção básica e escolas, por meio de ações de saúde bucal no ambiente escolar, como no Programa Saúde na Escola (PSE), pode ser uma estratégia preventiva efetiva contra a cárie dentária. Documentos técnicos e ações federais relatam a ampliação dessas atividades, com financiamento para insumos e procedimentos nas redes públicas. Ainda que a intensidade e a qualidade variem localmente, ressalta-se o papel mediador e protetor da escola sobre hábitos de higiene, acesso à prevenção e encaminhamento ágil para a rede de assistência à saúde^44^ ^,^ ^45^.

Algumas limitações devem ser reconhecidas, como o delineamento transversal, que restringe as inferências aos achados de associação, por não permitir relação de causalidade. Ademais, embora o instrumento SOHO-5 tenha sido aplicado tanto às crianças quanto aos seus pais ou responsáveis, deve-se considerar que, em crianças de 5 anos, a percepção sobre qualidade de vida pode ser parcialmente mediada pelo relato dos cuidadores, o que pode introduzir viés de percepção.

Destaca-se como ponto forte o foco em faixa etária específica (5 anos), o que reduz variações relacionadas ao desenvolvimento infantil, além do uso de instrumento validado para mensurar a QVRSB e metodologia adequada para avaliar a cárie, assegurando maior confiabilidade aos resultados. A análise integrada de fatores clínicos e sociodemográficos, incluindo a participação em programas de transferência de renda, reforça a relevância social dos achados e contribui para compreender a interação entre desigualdades sociais e saúde bucal.

Os resultados deste estudo mostram que as consequências clínicas da cárie na primeira infância, avaliadas pelo índice PUFA, impactam negativamente a qualidade de vida relacionada à saúde bucal em crianças brasileiras de 5 anos. Observou-se que essas desigualdades são complexas e multifatoriais, envolvendo determinantes sociais, econômicos, raciais e territoriais, com maior vulnerabilidade entre crianças indígenas, pardas e pretas, bem como aquelas de famílias de baixa renda ou residentes em regiões com menor cobertura de serviços odontológicos. Por outro lado, a frequência escolar apresentou efeito protetor, indicando que a integração entre atenção primária e ações de saúde bucal no ambiente escolar pode reduzir a carga da doença.

Nesse contexto, duas estratégias estruturantes do Sistema Único de Saúde (SUS) merecem destaque no enfrentamento das iniquidades em saúde bucal observadas neste estudo. A fluoretação das águas de abastecimento público, reconhecida como uma das medidas mais custo-efetivas de prevenção da cárie dentária, apresenta impacto significativo na redução da doença, sobretudo entre crianças de grupos socioeconomicamente mais vulneráveis^45^ ^,^ ^47^. Evidências nacionais indicam que descontinuidades na fluoretação e falhas no monitoramento dos teores de flúor contribuem para a persistência de desigualdades regionais e para maiores níveis de cárie não tratada na infância^46^.

Ademais, a adequada organização da Atenção Primária à Saúde, com a inserção efetiva das Equipes de Saúde Bucal (eSB) na Estratégia Saúde da Família, desempenha papel central na promoção da equidade em saúde bucal^48^. Contudo, em diversos municípios brasileiros, observa-se uma proporção inadequada entre equipes de Saúde da Família e eSB, o que sobrecarrega a atuação das eSB e limita o alcance das ações preventivas e educativas, especialmente entre crianças em idade pré-escolar. Essas fragilidades organizacionais comprometem a capacidade da atenção básica de atuar de forma oportuna sobre os determinantes sociais da cárie dentária e suas consequências clínicas mais severas.

Assim, o fortalecimento da fluoretação das águas com monitoramento adequado, aliado à ampliação e qualificação da atuação das eSB na Estratégia Saúde da Família, mostra-se essencial para a redução das consequências clínicas da cárie na primeira infância e de seus impactos sobre a qualidade de vida relacionada à saúde bucal^47^ ^,^ ^48^. Esses achados reforçam que a redução das desigualdades em saúde bucal depende da consolidação de políticas públicas estruturantes, capazes de enfrentar vulnerabilidades territoriais e sociais desde os primeiros anos de vida.
